# High-Capacity Spatial Structured Light for Robust and Accurate Reconstruction

**DOI:** 10.3390/s23104685

**Published:** 2023-05-12

**Authors:** Feifei Gu, Hubing Du, Sicheng Wang, Bohuai Su, Zhan Song

**Affiliations:** 1 Chinese Academy of Sciences, Shenzhen Institute of Advanced Technology, Shenzhen 518055, China; 2 The Department of Mechanical and Automation Engineering, The Chinese University of Hong Kong, Hong Kong 999077, China; 3 School of Mechatronic Engineering, Xi’an Technological University, Xi’an 710032, China

**Keywords:** spatial structured light, high capacity, pseudo-2D coding strategy, corner detection, 3D reconstruction

## Abstract

Spatial structured light (SL) can achieve three-dimensional measurements with a single shot. As an important branch in the field of dynamic reconstruction, its accuracy, robustness, and density are of vital importance. Currently, there is a wide performance gap of spatial SL between dense reconstruction (but less accurate, e.g., speckle-based SL) and accurate reconstruction (but often sparser, e.g., shape-coded SL). The central problem lies in the coding strategy and the designed coding features. This paper aims to improve the density and quantity of reconstructed point clouds by spatial SL whilst also maintaining a high accuracy. Firstly, a new pseudo-2D pattern generation strategy was developed, which can improve the coding capacity of shape-coded SL greatly. Then, to extract the dense feature points robustly and accurately, an end-to-end corner detection method based on deep learning was developed. Finally, the pseudo-2D pattern was decoded with the aid of the epipolar constraint. Experimental results validated the effectiveness of the proposed system.

## 1. Introduction

Three-dimensional reconstruction is one of the most important techniques in the research domain of computer vision, which has been widely applied for robotics, AR/VR, industrial inspection, human-computer interaction, and so on [1,2,3]. Different from temporal structured light (SL), spatial SL can achieve three-dimensional measurements with a single shot, which makes it an important branch in the field of dynamic reconstruction [4,5,6]. As a common technique for reconstruction, its accuracy, robustness, and density are therefore of vital importance. 

**Figure 1:**
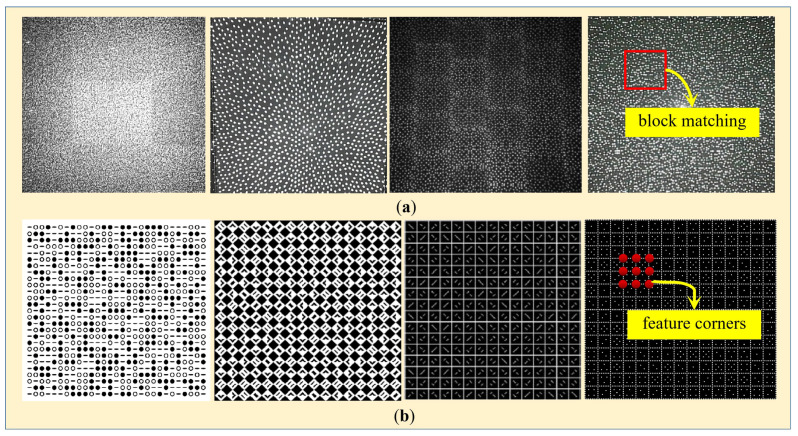
Some representative coding patterns in spatial SL. (**a**) **Speckle-coded SL.** From left to right are the speckle pattern used in Microsoft Kinect v1, Intel RealSense D435, Orbbec Astra Pro, and our previous work [8] respectively. (**b**) **Shape-coded SL**. From left to right are the 2D coding pattern designed in [12,13,14,15] respectively.

However, in spatial SL, there is a wide performance gap between dense reconstruction (but less accurate, e.g., speckle-coded SL) and accurate reconstruction (but often sparser, e.g., shape-coded SL). In speckle-coded SL, a speckle pattern is projected to attach textures onto object surfaces, and the reflected light is captured by one or two sensors [7,8,9]. Through the block-by-block matching of sensors, such as the well-known semi-global matching (SGM) algorithm, a high-density depth map can be realized. Yet, the accuracy of speckle-coded SL is limited due to block matching. Some representative speckle patterns are shown in Figure 1a. Generally, for these SL systems, only millimeter level accuracy can be achieved at a distance of 1.0 m. Contrary to this, in shape-coded SL [10,11,12,13,14,15], the speckles are substituted by different coding primitives, such as geometric shapes. The codeword of each feature point is determined by its values of itself, along with all adjacent elements surrounding it. In this case, by designing easy-to-detect feature points, such as corners, the reconstruction accuracy can be greatly improved. Yet, only sparse feature points can be reconstructed, which limits the spatial resolution greatly. Some representative shape-coded patterns are shown in Figure 1b.

To ensure high accuracy and spatial resolution at the same time, the coding capacity of shape-coded SL needs to be greatly improved. Currently, 2D matrix-like coding strategies are widely adopted in encoding the projected pattern, e.g., de Bruijn sequence [16], pseudorandom array [17], M-array [18], perfect map (PM) [19], and so on. Albitar et al. [12] generated a pattern based on the M-array with a coding capacity of 27 × 29, which used three different geometrical shapes, i.e., disc, circle, and dash, and the size of the coding window was 3 × 3. Yang et al. [18] improved the coding capacity to 27 × 36 with the same coding window by using six geometrical shapes, i.e., short lines, solid circles, hollow circles, triangles, stars, and diamonds. Tang et al. [13] generated a pattern based on the M-array with a coding capacity of 65 × 63, which used eight different geometrical shapes and a small coding window of 2 × 2. In our previous work [14], a pattern with a maximum coding capacity of 65 × 63 was also generated based on the M-array, which used eight different geometrical shapes, i.e., points with different arrangements, and a small coding window of 2 × 2. In another previous work of ours [15], a 36 × 48 pattern was designed based on the M-array to suit the VGA-level image capture device. Eight different geometrical shapes constituted by mahjong dots were designed and the size of the coding window was 2 × 2.

**Figure 2:**
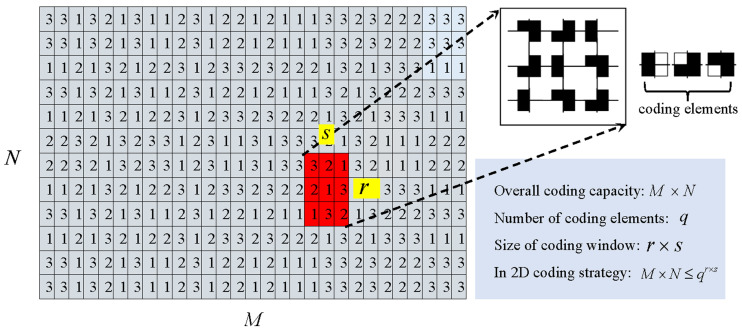
The overall coding capacity in 2D coding strategies is limited by the number of coding elements and the size of the coding window. (Reprinted/adapted with permission from Ref. [10]. April 2004, Elsevier”).

From the above research, it can be seen that in these 2D matrix-like coding strategies, the maximum coding capacity is theoretically limited [10]. To be more specific, the maximum coding capacity C (C=M×N) is determined by the number of coding elements q and the coding window’s size r×s: $M\times N\leq q^{r\times s}$, as shown in Figure 2. In this 2D matrix M×N, every sub-matrix r×s appears only once. In this case, a higher coding capacity can be achieved only by using more coding elements, or by enlarging the coding window’s size. However, both methods have their side effects. On one hand, the incorporation of more coding elements will inevitably make the decoding task more challenging. On the other hand, enlarging the size of the coding window will lead to a lower decoding accuracy, and additionally, occlusion and discontinuity of the scene will potentially lead to broken codewords. It is worth noting that this is also the reason why spatial SL has a much lower coding capacity compared with temporal SL. How to obtain a higher coding capacity with fewer coding elements and smaller coding windows is an interesting and meaningful research topic in the field of spatial SL.

To this end, this work developed a new pseudo-2D coding method and completed corresponding pattern decoding algorithms. The contributions of this paper are as follows. Firstly, a new pseudo-2D pattern generation strategy was developed, which can improve the coding capacity of shape-coded SL greatly. Then, to extract the dense feature points robustly and accurately, an end-to-end corner detection method based on deep learning was developed. Finally, the pseudo-2D pattern was robustly decoded with the aid of the epipolar constraint. The paper is organized as follows. Section 2 describes the main algorithms, such as the pattern design, corner detection, and feature decoding methods. Experimental results are given in Section 3, and some discussions and conclusions were drawn in Section 4 and Section 5, respectively.

## 2. Key Technology and Algorithm

### 2.1. Pseudo-2D Coding Method

The primary reason why the traditional 2D matrix-like coding strategy has theoretically limited the maximum coding capacity is that the r×s sub-matrix appears only once in the whole matrix. To overcome this limitation, we limited this constraint to a specific direction, for example, the image rows. Assuming the number of coding elements q and the coding window r×s, the detailed operation to generate a pseudo-2D matrix was as follows. 

Firstly, we generated a 1D pseudo-random sequence S. This was a typical multivariate pseudo-random sequences generation problem q≥2. A field with elements q forms a *Galois field* and is denoted by $GF\left(q\right):\;GF\left(q\right)=\left\{0,1,\ldots ,q-1\right\}$ [20]. An appropriate primitive polynomial h (x) should therefore be carefully chosen. Since fewer elements and smaller coding windows are required, the primitive polynomials in the *Galois field* m=2,3,4, q=3,4,5,6 are provided in Table 1. Here, A is a primitive element of $GF\left(q\right)$, and m is the size index of the coding window, with m=r×s. The length of the generated 1D pseudo-random sequence is $L\left(S\right)=q^{m}-1$.

Secondly, we created a coding sequence primitive $S^{\prime }$ from S. One way to do this is by using the first W entries of S on the first row of $S^{\prime }$, and the next W entries on the second row of $S^{\prime }$. In this way, a $S^{\prime }$ with the size of r×W was generated. $W=\left\lfloor L\left(S\right)/r\right\rfloor$. Here,   denotes the round-down operation. For example, assuming that q=4 coding elements are used, and the size of the coding window was 2×2, therefore r=2, s=2, m=4. Then, $L\left(S\right)=q^{m}-1=255$. In this case, the dimension of the generated pseudo-random sequence $S^{\prime }$ is r×W=2×127. Here, $S^{\prime }$ forms the coding sequence primitive of our whole pseudo-2D coding matrix. Other ways to generate $S^{\prime }$ include, but are not limited to, performing a simple horizontal cyclic shifting operation of $S^{\prime }$ to generate a new coding sequence primitive with a greater discriminating ability.

**Figure 3:**
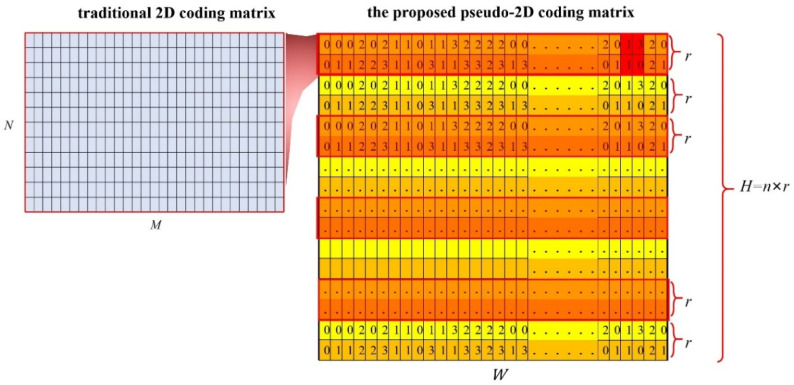
Schematic diagram of the proposed pseudo-2D coding method (taking r=2, s=2 as an example).

Thirdly, we generated the whole pseudo-2D coding matrix E. The final coding matrix was achieved by repeating $S^{\prime }$ several times along the direction of the image columns. The number of repetitions n was determined by considering the actual demand. Therefore, the dimension of the final E was W×H (H=n×r), and in other words, the coding capacity of E was W×n×r. To be clearer, the schematic diagram of the proposed pseudo-2D coding method is displayed in Figure 3.

In Figure 3, compared to the traditional 2D coding method, the proposed method rearranged the 1D pseudo-random sequence into a smaller height r instead of N. In this way, the coding capacity along the image rows can be greatly improved. Then, the whole coding capacity can be flexibly adjusted according to different needs by repeating the coding sequence primitive. There would be no need to use more coding elements or enlarge the coding window to improve the coding capacity as before. The traditional 1D coding method is a special case of the proposed pseudo-2D coding strategy with r=1. Indeed, a corresponding decoding method was required, which will be further elaborated in Section 2.2 and Section 2.3.

### 2.2. Detection of the Coded Feature Points

Extracting out the feature corners is the first procedure to decode a pattern [21]. In this work, the feature corners refer to the grid points that were formed by the horizontal and vertical lines. In an actual situation, the projected pattern will be modulated by the surfaces of target objects, which inevitably would lead to the problem of fuzzy features, structural deformation, and texture fusion. These phenomena are more prominent in the proposed high-capacity SL pattern due to the distribution of feature points being more and denser. In this case, traditional template matching methods have their obvious shortcomings of low robustness and may likely fail in problem areas [15,21]. To therefore improve the robustness of corner detection, an end-to-end convolutional neural network (CNN) that can classify and locate the feature corners simultaneously and directly from the input images is highly desired. 

**Figure 4:**
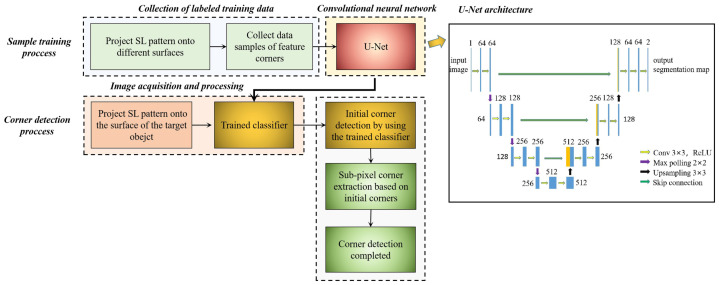
Route chart of the feature corners’ detection method based on the U-Net.

Among all widely used CNNs, U-Net [22] has been verified to have a superior performance in classification tasks since being developed. The advantage of using U-Net is that it can work with very few training images and yields more precise segmentation. Here, we introduced U-Net in this work to transform the problem of feature detection into a semantic segmentation problem. The route chart of our method is displayed in Figure 4.

(1) Collection of training Samples.

**Figure 5:**
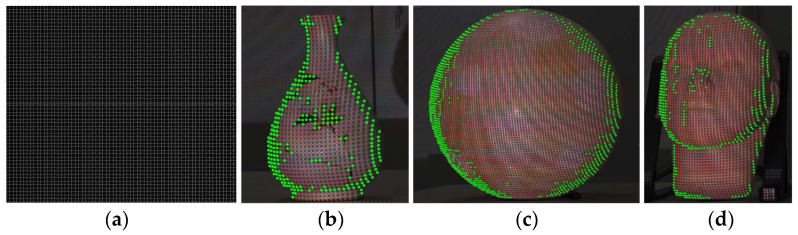
The projected SL pattern and some examples of the grid corners’ labeling results: (**a**) the projected SL pattern; (**b**) the corners’ labeling results of a vase; (**c**) the corners’ labeling results of a ball; (**d**) the corners’ labeling results of a face model. (Red dots represent automatic labeling results, and green dots represent manual labeling results).

We projected the SL pattern as shown in Figure 5a onto the surfaces of different objects. The labeling of the feature corners is an arduous task as there are thousands of them in a single image. Therefore, we combined manual and automatic methods to lighten the workload. First, we detected corners by using the Gaussian-cross module we developed in [15], which was then put in Equation (1). In this way, most corners beyond the problem areas were detected automatically. Then, for the corners that were difficult to detect, we labeled them one-by-one manually. Figure 5b–d shows several of the labeling results. It can be observed that all the corners that were present on the surfaces have been labeled successfully.

(1) $$\psi (x,y)=\sum_{i=0}^{2w}\sum_{i=0}^{2w}\underset{the\;input\;sample\;image}{\underbrace{I(x+i-w,y+j-w)}}\cdot \underset{the\;Gaussion\;model}{\underbrace{\overset{T(x,y)=\frac{1}{2\pi \sigma^{2}}e^{-\frac{{\left(x-r\right)}^{2}+{\left(y-r\right)}^{2}}{2\sigma^{2}}}}{\overbrace{T(x,y)}}}}\cdot \underset{the\;cross\;model}{\underbrace{\overset{C(x,y)=\left\{\begin{array}{cc} 1 & |x|<w_{x}/2\&|y|<w_{y}/2\\ 0 & \;if\;else \end{array}\right.}{\overbrace{C(x,y)}}}}$$

(2) End-to-end corner detection with U-Net.

**Figure 6:**
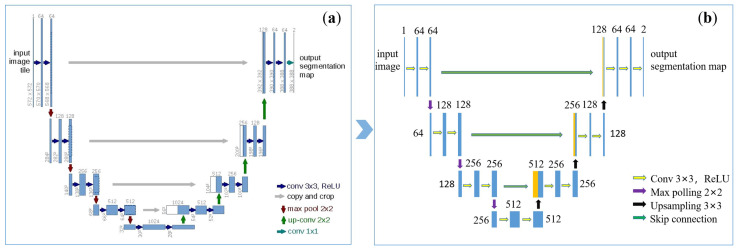
The U-Net architecture. (**a**) Original version; and (**b**) a simplified version adopted in this work.

In the above sample collection process, 30 images with labeled ones have been collected. A total of 20% of them were chosen as the validation set, while the others were designated as the training set. Then, we expanded the training set through data augmentation, such as with Gaussian noise, small affine transformation, random white/black lines, and so on, to achieve a higher discriminating power. Next, we fed the augmented training data to the network, as displayed in Figure 6. Note that the feature corners in this work are rather simple and small, and normally occupy a dozen to dozens of pixels in the captured images. In this case, a too-deep network is prone to overfitting. Therefore, the original U-Net structure was clipped to have a simplified version as shown below.

**Figure 7:**
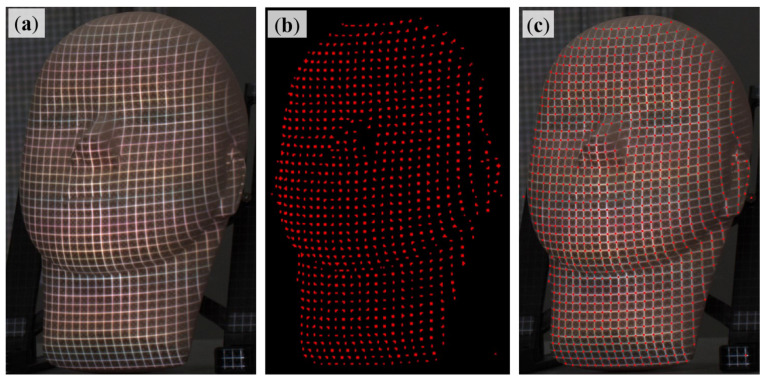
One example of grid corners’ detection results by U-Net. (**a**) The input captured image; (**b**) the output segmentation map; and (**c**) initial corner detection results.

The network structure consists of four parts: the image input layer, the down-sampling part, the up-sampling part, and the image output. The down-sampling part consists of three main blocks for feature extraction, with each block consisting of two 3 × 3 convolutions, and the activation function (relu) was added following the convolution. Specific details can be referred in [22]. Finally, a segmentation model for end-to-end corner detection was obtained, as shown in Figure 7b.

In the output segmentation map as shown in Figure 7b, each bump indicates the potential position of a feature corner. The bump may contain a dozen to dozens of pixels, with each pixel being assigned a prediction value. A higher prediction value means that it is more likely to be close to the center of the feature corner. Therefore, the initial position of each feature corner was obtained by calculating the square-weighted centroid of each bump. Taking one bump as an example, assuming that the number of effective pixels in this bump is *b*, the prediction value of each pixel $\left(x_{i},y_{i}\right)$ in this bump would be $I(x_{i},y_{i})\;(i=1,2,\ldots ,b)$. Therefore, the square weighted centroid of this bump is:

(2) $$x_{0}=\frac{\sum_{i=1}^{b}x_{i}\cdot I^{2}(x_{i},y_{i})}{\sum_{i=1}^{b}I^{2}(x_{i},y_{i})},y_{0}=\frac{\sum_{i=1}^{b}y_{i}\cdot I^{2}(x_{i},y_{i})}{\sum_{i=1}^{b}I^{2}(x_{i},y_{i})}$$

**Figure 8:**
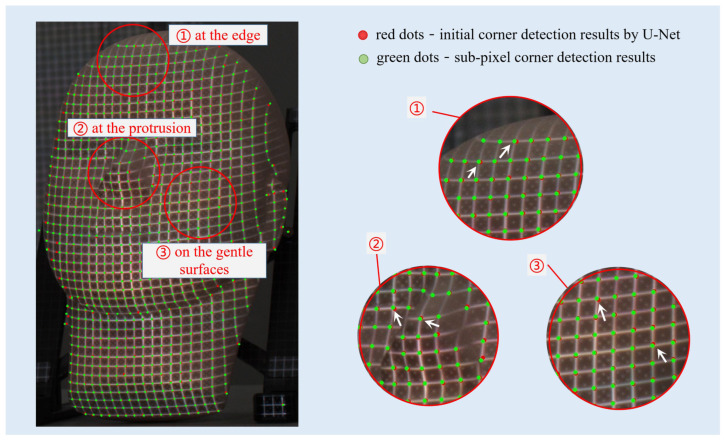
One example of grid corners’ detection results.

Here, $p(x_{0},y_{0})$ is the position of the feature corners. An example of initial corner detection results based on Equation (2) is shown in Figure 7c. It can be seen that the results are in good agreement with the raw image. However, it is also worth noting that the detected corners were greatly influenced by the accuracy of data labeling. U-Net was designed for segmentation. In segmentation tasks such as biomedical images, the location accuracy of each dividing curve for different regions is not that important. But in this work, the localization accuracy of feature corners directly determines the final measurement accuracy. Therefore, a high-accurate sub-pixel corner detection procedure is necessary and was applied here. Based on the initial corners as shown in Figure 7c, a non-maxima suppression method [23] was adopted. The sub-pixel-level position $\hat{p}(x_{0},y_{0})$ can be achieved by:

(3) $$\hat{p}(x_{0},y_{0})=\underset{p}{\min}\sum_{-w\leq i\leq w}\sum_{-w\leq j\leq w}{\left\{g_{p(x_{0}+i,y_{0}+j)}^{T}[p(x_{0}+i,y_{0}+j)-p(x_{0},y_{0})]\right\}}^{2}$$

where $g_{p(x_{0}+i,y_{0}+j)}^{T}$ denotes the gradient information of $p(x_{0}+i,y_{0}+j)$ against $p(x_{0},y_{0})$. Some results are displayed in Figure 8. It can be observed that U-Net can help mark potential corner positions correctly and robustly, and the non-maxima suppression method aids in detecting them accurately.

### 2.3. Decoding of the Pseudo-2D Coding Pattern

**Figure 9:**
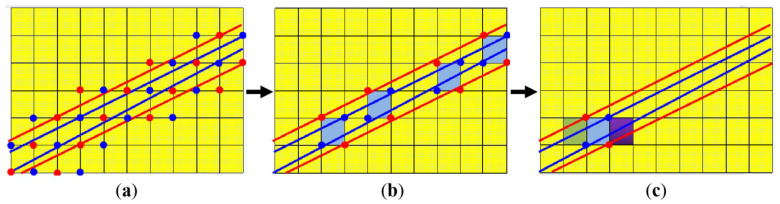
Diagram of the decoding process. (**a**) The search for all candidate matching blocks based on the epipolar constraint, (**b**) coarse matching based on the judgment of blocks’ codewords, (**c**) fine matching based on the constraint of the coding window.

Given the accurate feature points, 3D reconstruction can be accomplished after decoding. In our previous work [15], a simplified-ResNet model was introduced to accomplish the block recognition task, which achieved a rather high recognition accuracy of 97.98%. Therefore, this work adopted the same method. More information can be referred in [15]. As we now have accurate feature points, and the tool to recognize any pattern blocks, the codeword of each feature point should then be subsequently determined to accomplish stereo correspondences. Therefore, a ‘coarse-to-fine’ strategy guided by epipolar constraint was adopted to filter out false matching features and achieve correct correspondences. The detailed decoding procedure can be referred to Figure 9.

Firstly, for the four feature points of each block on the image plane of the projector (IPP), we calculated their corresponding epipolar lines on the image plane of the camera (IPC), as illustrated in Figure 9a, and found all the candidate counterparts that satisfied:

(4) $$d(x_{c},Fx_{p})<\delta$$

where $\left(x_{c},x_{p}\right)$ denotes the feature points on IPP and IPC, respectively, and δ denotes the distance error threshold of the epipolar constraint, which was determined by referring to the average value of the forward epipolar distance errors in the IPC obtained from the calibration process.

Secondly, we screened out all the candidate blocks that kept the same codeword with the reference block in the IPP, as shown in Figure 9b.

Lastly, we determined the best matching block by judging its neighbor blocks. As shown in Figure 9c, the uniqueness in the coding window size of, e.g., 1 × 3, helped retrieve the unique block correspondence between the IPP and the IPC. Naturally, the stereo correspondences of the four vertices of the block are simultaneously and uniquely matched. After matching all the feature points, 3D reconstruction was carried out smoothly.

## 3. Experiments and Results

**Figure 10:**
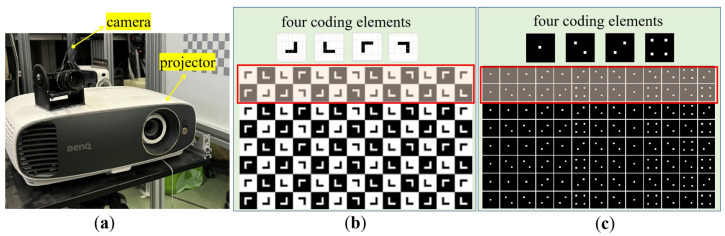
Experimental setup and two different pseudo-2D SL patterns. (**a**) Experimental setup; (**b**) part of the first pseudo-2D pattern with four coding primitives of ‘L’; and (**c**) part of the second pseudo-2D pattern with four coding primitives of mahjong dots.(Inside the red rectangular frame in Figure 10b,c is the designed coding sequence primitive).

The prototype of the proposed SL system is displayed in Figure 10a. A camera of PointGrey BFS-U3-120S4C-CS was used, which is of the resolution of 4000 × 3000, and can work at a frame rate of up to 31 fps. The pixel size of the camera was 1.85 μm. The projector used was a DLP projector BENQ w1700s, which is of the resolution of 3840 × 2160, and has a projection ratio (i.e., the ratio of the projector’s projection distance against the projected pictures’ width) of 1.47~1.76:1. The calibration algorithm in [24] was applied to calibrate the SL system accurately and the results are displayed in Table 2. 

Two SL patterns with different geometric shapes were designed based on the proposed pseudo-2D coding strategy. Both patterns were generated by using the primitive polynomial *h*(x) = x^4^ + x^2^ + Ax + A^2^, with four coding elements and a coding window of the size of 2×2. In the first pattern, the coding elements were designed as square blocks with embedded geometrical shapes, and the square blocks were specially colored with a black or white background, which thereby comprises a typical checkerboard pattern. The embedded geometric shapes adopted a simple ‘L’ shape with four different rotation angles ($0^{{}^{\circ}},\;90^{{}^{\circ}},\;180^{{}^{\circ}}$ and $270^{{}^{\circ}}$), as shown in Figure 10b. In the second pattern, speckle dots in different distributions were tucked into the blocks that were formed by a series of horizontal and vertical lines, as shown in Figure 10c. In both patterns, the grid corners formed by the horizontal and vertical lines were taken as the main feature points of SL. One of the irreplaceable advantages of these feature corners is that they can be extracted in sub-pixel precision. Based on the coding strategy in Section 2.1, given the number of coding elements (q=4) and the size of the coding window (m=r×s=2×2), the dimension of the pseudo-2D sequence ***S’*** was $r\times W=r\times \left\lfloor L(S)/r\right\rfloor =r\times \left\lfloor (q^{m}-1)/r\right\rfloor =2\times 127$. Afterward, repeating ***S’*** by n times, resulted in the pattern with a theoretical maximum coding capacity of n×r×W=n×2×127 being generated. In this work, the value of *W* was empirically set to be 65. Therefore, the whole coding capacities of both patterns are 16,510, which have been greatly improved compared with all previously reported work, as far as we know.

To test the proposed algorithms comprehensively, several experiments were conducted. First, the performance of the proposed end-to-end corner detection method was tested and compared with the traditional methods. Then, the measurement accuracy of the system was evaluated at different distances and compared with other market-available SL cameras. Afterward, surfaces with rich colors and complex textures were selected to test the robustness of our system.

### 3.1. Performance Evaluation of the Developed End-to-End Corner Detection Algorithm

One of the main contributions of this work is the development of an end-to-end corner detection method based on deep learning. Therefore, the performance of it was first evaluated. Here, the traditional method that was based on the local template matching algorithm as in [15] was used as a comparison. The details of this algorithm can be referred to [15].

#### 3.1.1. Performance w.r.t. (with Respect to) the Noise Level

**Figure 11:**
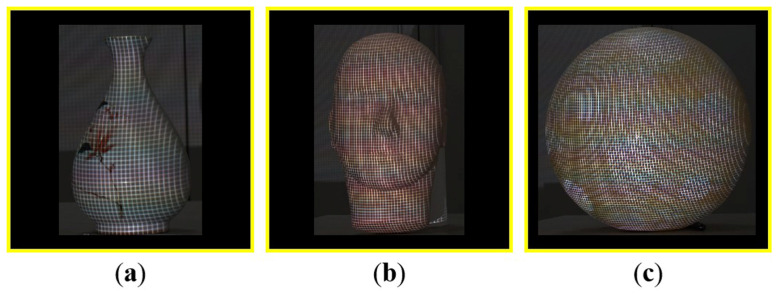
Three target objects. (**a**) A vase, (**b**) a face model, and (**c**) a ball.

**Figure 12:**
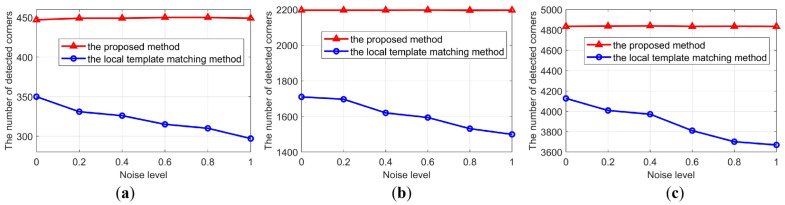
Corner detection data under different noise levels of the (**a**) vase, (**b**) face model, and (**c**) the ball, respectively.

In this experiment, three different objects were used. One was a vase with textured surfaces, another was a face model with large curvature-changing surfaces, and the last was a ball with smooth surfaces, as shown in Figure 11. To test the robustness of the developed end-to-end corner detection method, Gaussian noise with the mean value of 0 and the standard deviation of σ was added to the raw images. Considering the normal noise in a practical measurement situation, we varied σ from 0 to 1.0. The corner detection results under different noise levels of the proposed method were shown in Figure 12. The results of the traditional local template matching algorithm [15] was provided for comparison.

As can be seen in Figure 12, for these three objects, with the increase in noise level, the performance of the traditional image processing method [15] was reduced. However, the proposed method demonstrates a stable performance and robustness to noise. For the traditional local template matching method, when σ = 1, the number of successfully detected corners of the three objects decreased by 17.8%, 14.1%, and 12.5%, respectively, compared with the noise-clean situation. However, for the proposed method, the results were only 1~3 corner points apart.

#### 3.1.2. Performance w.r.t. the Density of the Feature Points

**Figure 13:**
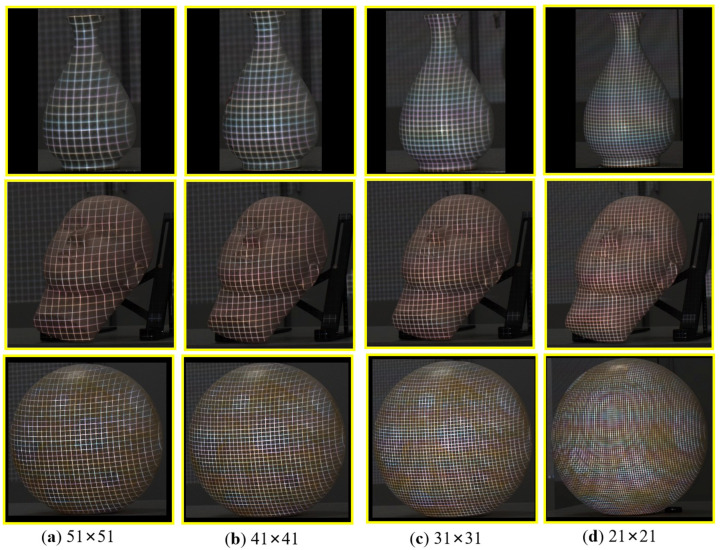
Projected SL patterns displayed in Figure 5a with different coding densities onto the surfaces of a vase, a face model, and a ball, respectively.

To further validate the performance of the proposed method, we compared it with the traditional image processing method [15] in the case of different coding densities. We changed the coding density of the pseudo-2D pattern by changing the size of the pattern blocks. Here, the pattern blocks with the size of 51 × 51 pixels, 41 × 41 pixels, 31 × 31 pixels, and 21 × 21 pixels were chosen, respectively. The smaller the block size, the greater the coding density. Three target objects, a vase, a face model, and a ball were used. The coding SL pattern in Figure 10b was used, and the captured images with different coding densities are displayed in Figure 13.

The quantitative corner detection results of the three objects based on the traditional image processing method and the proposed method were comparatively displayed in Table 3. The number of successfully detected corners was used as an index to evaluate the performance of these two different methods. In Table 3, the growth rate of the number of corner extraction by our method and the traditional image processing method was expressed as “Ratio”.

Several conclusions were made from these results. First, in all the target surfaces, the number of detected corners improved greatly using the proposed method, rather than the traditional method. For example, for the vase with a pattern density of 21 × 21 pixels, the corner detected by our method was 26.3% higher than that with the traditional method. Second, our method demonstrates a superior robustness against the changing of the coding densities. Taking the spherical data as an example, although the projected corners have been very dense with the pattern density of 21 × 21 pixels, our method can detect most of the features correctly. Last, the advantages of our method are more obvious when dealing with the problem areas, such as the edges, surfaces with rich textures, or large curvature changes. For the ball with the pattern density of 21 × 21 pixels, the corner detected by our method was 17.1% higher than that by the traditional method. By contrast, this data were as high as 27.8% for the face model. 

### 3.2. Accuracy Evaluation of the Developed System

**Figure 14:**
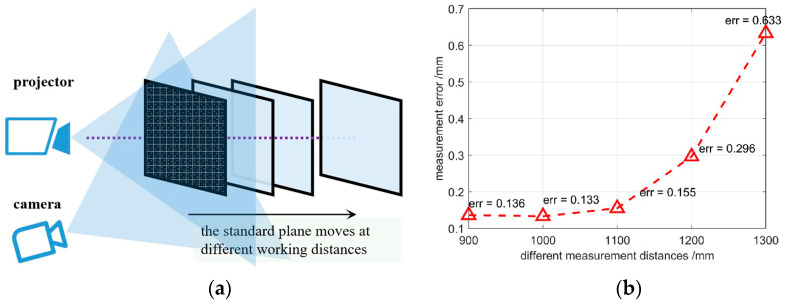
Reconstruction accuracy of the proposed system. (**a**) Schematic diagram of the accuracy evaluation experiment; and (**b**) reconstruction errors in different working distances.

Reconstruction accuracy of the developed spatial SL with a high-capacity pattern was examined. A standard plane of size 50 × 50 cm was utilized as the target object and the machining accuracy of it was up to ±5 µm. The plane was put at different working distances from 90 cm to 130 cm against the projector with a step of 10 cm. The plane was reconstructed, and its standard deviation was taken as the precision evaluation index. The results are displayed in Figure 14. At working distances of 1.0 m, the reconstruction error was as low as 0.133 mm, which achieves submillimeter accuracy. When the working distance increased to 130 cm, the accuracy markedly decreased to 0.633 mm. Two main reasons may have led to this phenomenon. The first could have been the triangular structure of the SL system, in which the accuracy decreases with increasing measuring distances. The second reason could have been the depth of field of the DLP, which was rather small once the focal length of the lens was fixed. When the distance reached 130 cm, the projected patterns were already blurred.

### 3.3. Reconstruction of Surfaces with Rich Textures

**Figure 15:**
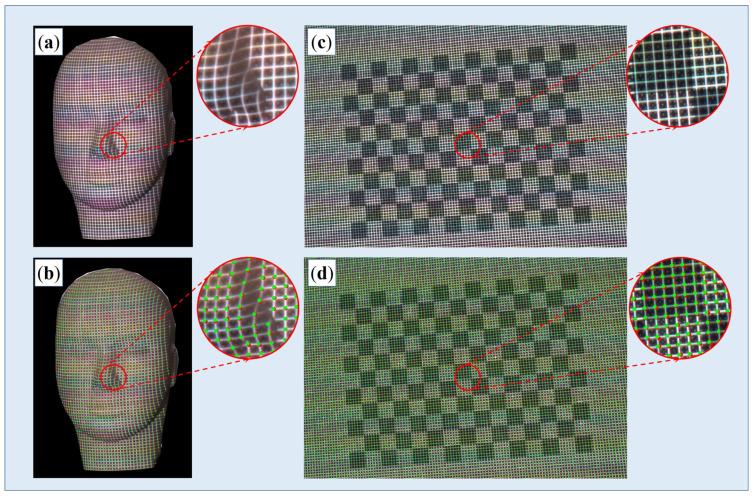
Target objects with complex textures. (**a**,**b**) Raw image and corner detection results of a face model with varying geometrical textures, respectively; (**c**,**d**) Raw image and corner detection result of a chessboard with varying color textures, respectively.

**Figure 16:**
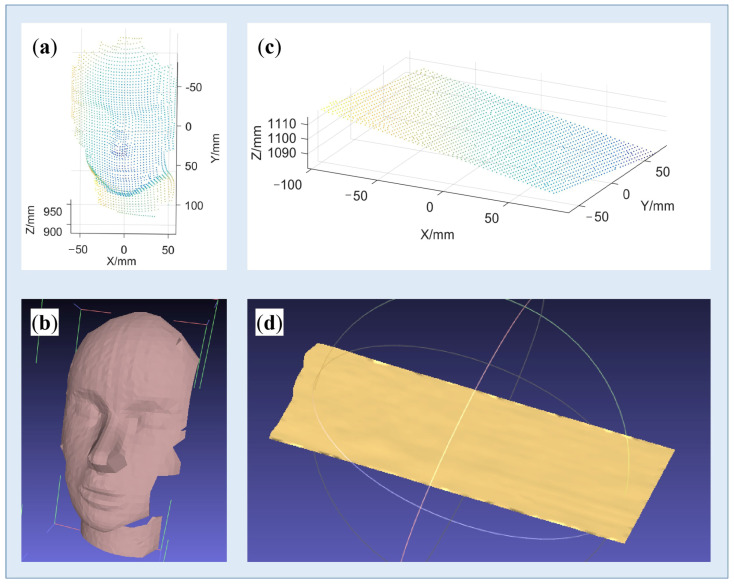
Reconstruction results. (**a**,**b**) Point cloud and 3D model of the face model; (**c**,**d**) Point cloud and 3D model of the chessboard.

As mentioned above, objects’ textures, including both geometrical texture and color texture, bring great challenges in pattern decoding. In this part, two different target objects were selected. One was a face model with complex geometrical textures, and another was a plane with complex color textures, as shown in Figure 15a,c. Corresponding results of corner detection are shown in Figure 15b,d, respectively. Reconstructed point clouds and 3D models are displayed in Figure 16. It can be observed that the proposed method works well on complex surfaces.

### 3.4. Reconstruction of Surfaces with Large Mutations

**Figure 17:**
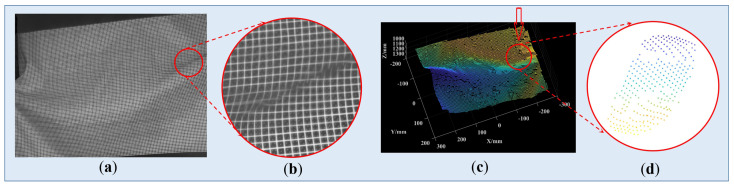
Reconstruction results of the deformed surfaces based on the SL pattern in Figure 5a. (**a**) the captured frame; (**b**) the enlarged details of (**a**); (**c**) the reconstructed results; and (**d**) the enlarged details of (**c**).

To further validate the robustness of the proposed method, a piece of paper was shaken to generate various and large morphological mutations. The proposed system shot the dynamic scenes and recovered the 3D morphology of the deformed surfaces. As the projected pattern was fixed, only the frame rate of the camera needed to be adjusted. Here, we made it work at the frame rate of 31 fps. We shook the paper to create different deformations and captured 35 frames in total. The captured images and reconstructed results can refer to the videos we provided in the attached file. To save some space, only one of the frames and its reconstruction result were displayed in Figure 17.

From Figure 17a,b, it can be seen that in the regions with an abrupt surface gradient, the blocks of the SL pattern were twisted, or even broken. For small and moderate mutations, our system was found to work rather well. However, for serious and large mutations, the pattern blocks were broken, indicating that they cannot be successfully extracted and decoded, meaning that the related 3D data was lost. It should be noted that this is a recognized defect of spatial SL. In fact, by adopting denser coding corners with our high-capacity pattern, this problem can be alleviated to some extent, but the problem still exists. The processing of broken code words is an important research direction of spatial SL, which has been planned to be studied in further depth in our future work.

## 4. Discussions

Spatial SL technology can achieve high-precision reconstruction from a single image and is one of the important 3D vision technologies. However, it has not been practically applied compared to the temporal SL and is mainly limited by the traditional 2D matrix-like coding strategies, resulting in fewer reconstructable feature points. This paper proposed a new pseudo-2D coding method that greatly increases the number of spatial coding points by reducing the coding dimensions. Taking the common settings of the *q* = 4 coding elements and the coding window of *r* × *r* = 2 × 2 as an example, the traditional 2D matrix-like coding strategies can only generate a pattern with a theoretical maximum coding capacity of 255, while the proposed pseudo-2D coding method can generate a pattern with the maximum coding capacity of *n* × *r* × *W* = *n* × 254 (where n is the number of repetitions). It is worth noting that the increase in coding density has also led to an increased decoding difficulty, especially in the extraction of dense feature points. To address this issue, this paper proposes an end-to-end deep learning method that enhances the robustness compared to the traditional template matching methods, especially in the problem areas, such as the edges, surfaces with rich textures, or large curvature changes.

However, several disadvantages and limitations of the proposed technique remain. For example, as the coded feature points are still discrete, there may be code interruption issues for objects with surface discontinuities or large morphological mutations. Additionally, this system is relatively bulky due to the use of a high-resolution DLP projector. To make it more portable and compact, we are considering using diffractive optical elements for projection in the future.

## 5. Conclusions

This paper proposed a high-capacity spatial SL system to achieve robust and accurate reconstruction. By developing a new pseudo-2D pattern generation strategy, the theoretical maximum coding capacity in shape-coded SL has been greatly improved compared with the traditional 2D-matrix-like coding strategies. By introducing an end-to-end corner detection method based on deep learning, the robustness of spatial SL has been improved, which we believe can promote this technique’s practical application in precise measurements in the near future. Note that through this work, all critical steps in the procedure of pattern decoding, e.g., corner detection and block recognition, have been solved by deep learning, rather than by conventional image processing methods, which can guarantee a higher robustness in practical applications. We plan to transfer the proposed system into a miniaturized system in our next step. The designed high-capacity pseudo-2D SL pattern will be etched onto the diffractive optical elements and then projected. In addition, how to achieve high-precision measurements in the case of short baselines will be researched.

## Figures and Tables

**Table 1:** Primitive polynomials in *the Galois field*.

*m* (*m* = *r* × *s*)	*q* = 3	*q* = 4	*q* = 5	*q* = 6
2	x^2^ + x + 2	x^2^ + x + A	x^2^ + Ax + A	x^2^ + x + A
3	x^3^ + 2x + 1	x^3^ + x^2^ + x + A	x^3^ + x + A	x^3^ + x + A
4	x^4^ + x+ 2	x^4^ + x^2^ + Ax + A^2^	x^4^ + x+A^3^	x^4^ + x + A^5^

**Table 2:** Calibration parameters of the SL system.

	Focal Length/Pixels	Principal Points/Pixels	Lens Distortion Coefficients
Camera	(8980.67, 8975.48)	(1991.58, 1511.24)	(−0.077,0, 0, 0, 0)
Projector	(6805.51, 6797.03)	(1942.82, 2946.19)	(−0.009, 0, 0, 0, 0)
Translation vector T/mm: (156.79, −108.33, 41.52)			
Rotation vector om: (0.18, −0.17, 0.08)			

**Table 3:** Accuracy comparison between the traditional method and the proposed method with different coding densities.

Different Sizes of Pattern Blocks/Pixels			51 × 51	41 × 41	31 × 31	21 × 21
Number of detected feature corners	Vase	Ground truth	135	251	449	845
		Traditional method	110	197	350	661
		The proposed method	134	250	447	835
		Ratio	↑21.8%	↑26.9%	↑27.7%	↑26.3%
	Face model	Ground truth	285	525	903	2218
		Traditional method	224	410	701	1710
		The proposed method	285	523	902	2196
		Ratio	↑27.2%	↑27.6%	↑28.7%	↑27.8%
	Ball	Ground truth	643	1103	1932	4898
		Traditional method	551	935	1620	4128
		The proposed method	642	1101	1926	4834
		Ratio	↑16.5%	↑17.8%	↑18.9%	↑17.1%

## Data Availability

Data is contained within the article.
